# Supplementary material for: Cyclosporine a directly affects human and mouse b cell migration in vitro by disrupting a hIF-1 αdependent, o2 sensing, molecular switch
Source: BMC Immunol. 2020 Mar 18;21:13. doi: 10.1186/s12865-020-0342-8 (PMC7079363; doi:10.1186/s12865-020-0342-8)
Supplement: Supplementary file 1 — Additional file 1 Supplementary figures. [file 12865_2020_342_MOESM1_ESM.pdf]

Additional Files

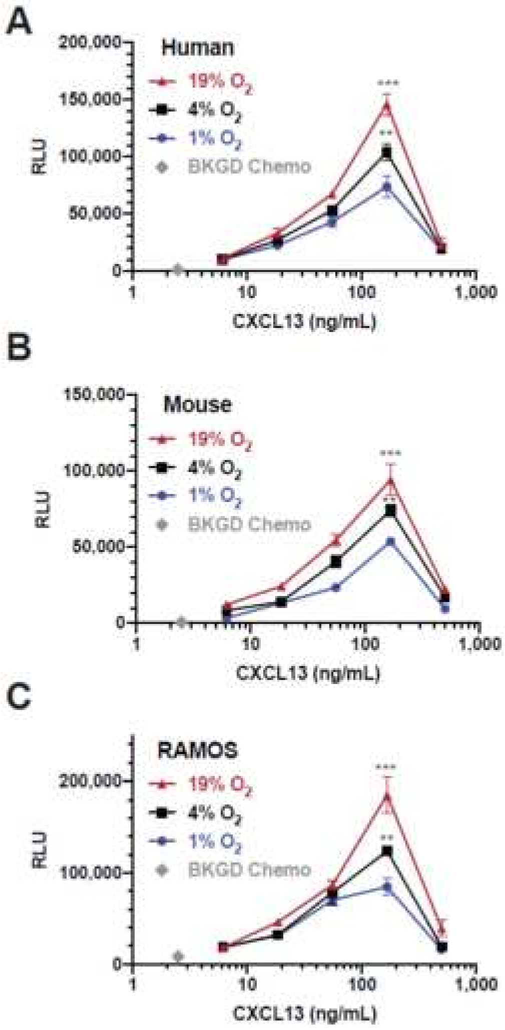

Figure 1 Chemotactic differences are recapitulated with CXCR5/CXCL13. Human (**a**), mouse (**b**) or RAMOS B cells (**C**) were incubated at the indicated O_2 levels for 24 hours. After the incubation, cells were harvested and assessed for CXCL13 chemotactic activity. Shown is representative experiment from 3 independent experiments. ***P<0.001; **P<0.01

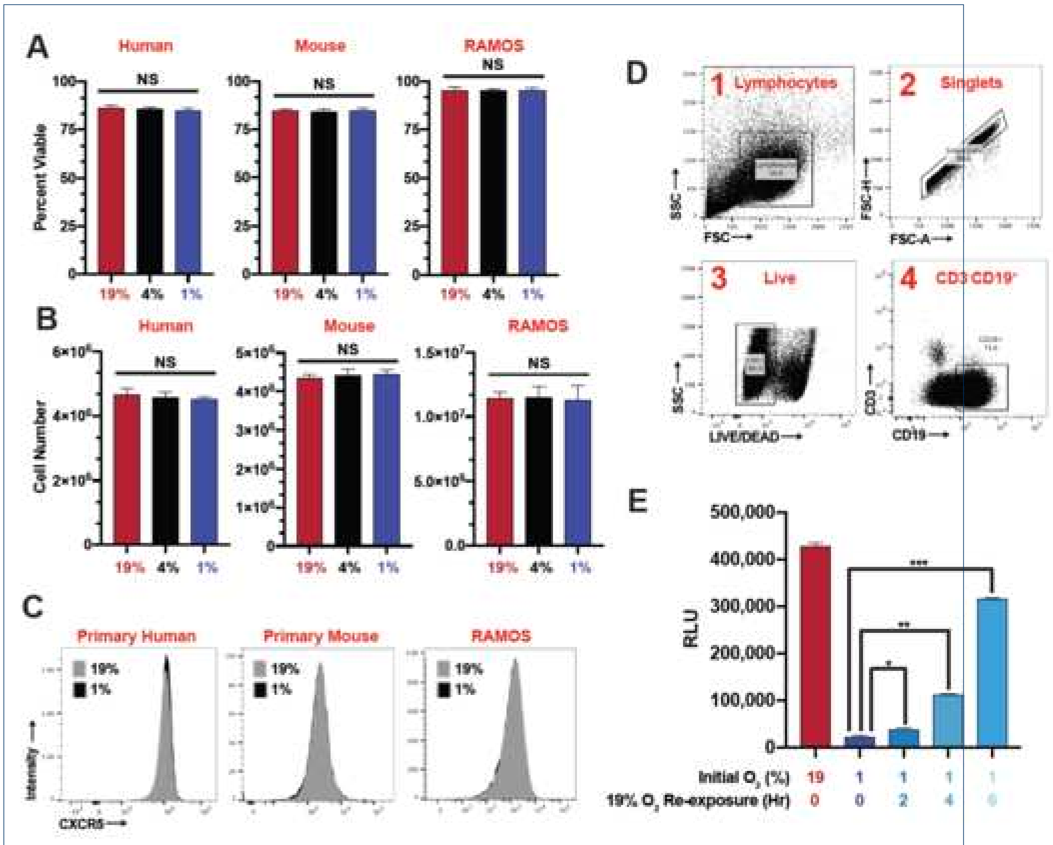

Figure 2 Chemotactic differences are not due to changes in cellular viability, proliferation or chemokine receptor surface expression. Human, mouse or RAMOS B cells (5 x 10^6 cells per condition at 5 x 10^5 cells/mL, in triplicate) were incubated at the indicated O_2 levels for 24 hours. After the incubation, cells were harvested and assessed for (**a**) viability (trypan blue exclusion), (**b**) proliferation by assessing total cell numbers or (**b**) surface CXCR5 levels assessed by flow cytometry. (**c**) CXCR5 surface staining does not significantly change with changing O_2 levels. (**d**) Sequential gating strategy. (**e**) RAMOS cells were incubated for 24 hours at 19% or 1% O_2 levels. After 24 hours, RAMOS cells incubated at 1% O_2 were re-exposed to 19% O_2 for 2, 4 or 6 hours and then subjected to our chemotaxis assay. Shown is representative experiment from 3 independent experiments. ***P<0.001; **P<0.01; *P<0.05; NS = not significant.

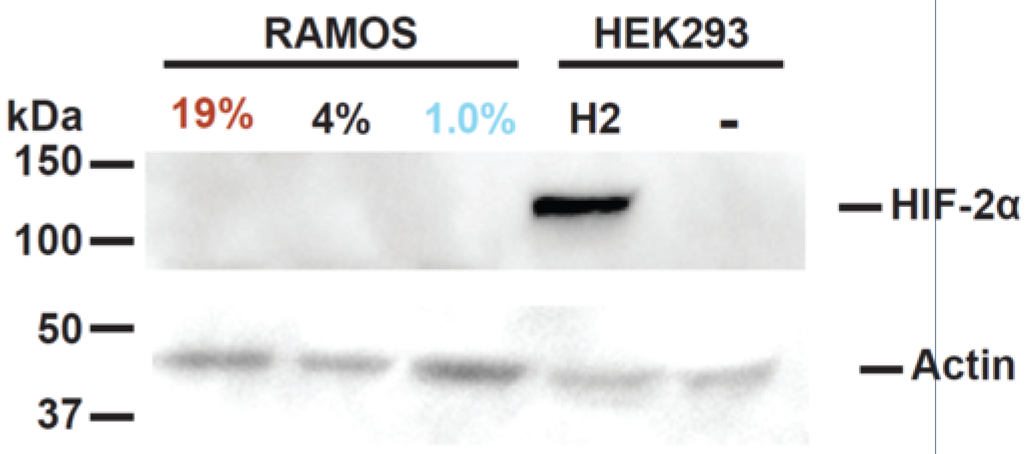

Figure 3 HIF-2α stabilization is not observed in RAMOS B cells after exposure to low O_2 levels. RAMOS cells were incubated at the indicated O_2 levels for 24 hours. After the incubation, cells were harvested, lysates were prepared and westerns performed to measure HIF-2α levels with actin being used as a loading control. Lysates from HIF-2α over-expressing (H2) or non-transfected (-) HEK293 cells, were used as positive and negative controls, respectively. Shown is a representative blot from 3 independent experiments.

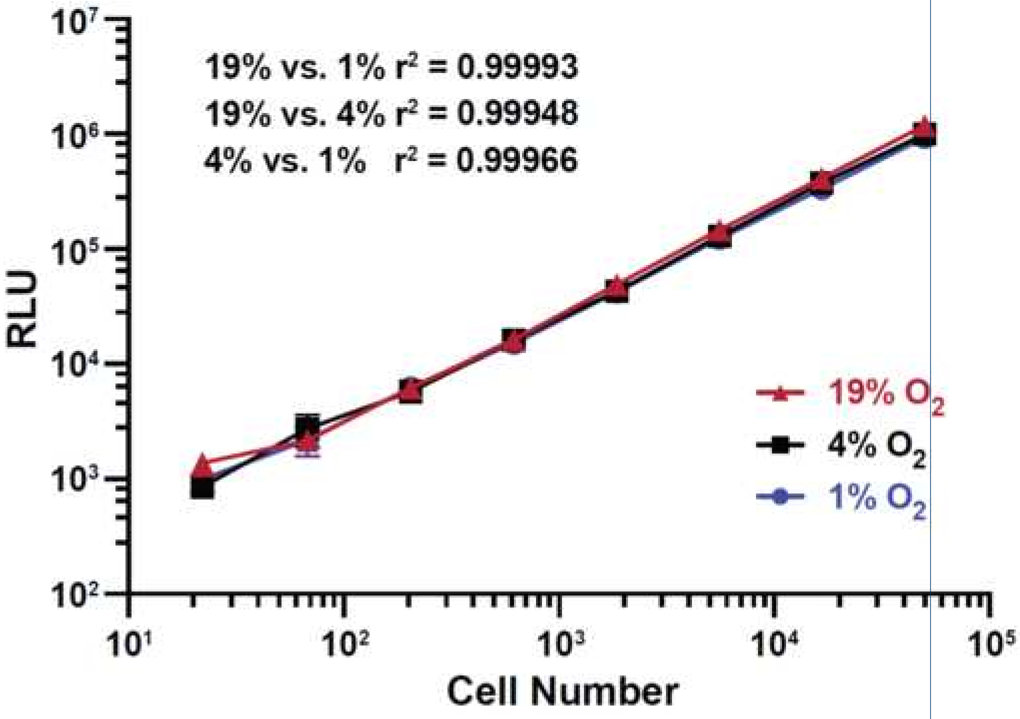

Figure 4 A direct correlation exits between cell numbers and RLU readout regardless of O_2 levels. RAMOS cells (5 x 10^6 cells per condition at 5 x 10^5 cells/mL, in triplicate) were incubated at the indicated O_2 levels for 24 hours. After the incubation, cells were harvested and counted. Cells were then plated in triplicate well in 96 well plates at the indicated cell numbers. Plates were then incubated at the original O_2 levels for an additional hour and luminescence was then assessed to mimic the conditions of the chemotaxis assay. assessed for (**a**) viability (trypan blue exclusion), (**b**) proliferation by assessing total cell numbers or (**c**) surface CXCR4 levels assessed by flow cytometry. Shown is representative experiment from 3 independent experiments. Pearson correlation coefficients were calculated using Prism software (GraphPad Software, San Diego, CA; Mac version 8.0.2)
